# Roles of stay-green (SGR) homologs during chlorophyll degradation in green plants

**DOI:** 10.1186/s40529-020-00302-5

**Published:** 2020-09-23

**Authors:** Baozhen Jiao, Qingwei Meng, Wei Lv

**Affiliations:** State Key Laboratory of Crop Biology, College of Life Science, Shandong Agricultural University, Daizong Street, Tai’an, 271018 Shandong People’s Republic of China

**Keywords:** Chlorophyll degradation, Stay-green (SGR) homologs, Degreening

## Abstract

Chlorophyll (Chl) degradation is one of the most obvious signs of leaf senescence and fruit ripening. Stay-green (SGR) homologs that can remove magnesium from Chl *a* are the most important components in Chl degradation pathway in green plants. SGR homologs are not only universally involved in Chl breakdown during the senescence of green organs, but also play crucial roles in other organs during plant growth and development, such as fruit mature and nodule development. In this review, we focus on the diverse functions of SGR homologs in plant growth and development. A better understanding of SGR would be helpful for providing a theoretical basis for further illustrating the regulatory mechanism of SGR homologs.

## Background

In plants, chlorophyll (Chl) molecules, includes Chl *a* and Chl *b*, are the components of the photosynthetic complex, playing crucial roles in absorption, transmission, and transformation of light energy (Hortensteiner 2009). In autumn, plant leaves generally change in color from green to yellow or red as a result of Chl breakdown combined with carotenoid retention or anthocyanin accumulation (Matile et al. 1999). Due to Chl degradation, degreening has become the most typical symptom during leaf senescence, fruit ripening, and seed maturation. Besides this, the released nutrients from Chl degradation would be recycled to fast-growing vegetative tissues and reproductive organs, thus facilitates nutrient remobilization (Vom Dorp et al. 2015). In addition, Chl degradation is coupled with the breakdown of light-harvesting complexes, and proper degradation of Chl molecules would protects photosynthetic apparatus from advese growth conditions (Hortensteiner 2006; Li et al. 2017). Therefore, the process of Chl degradation plays a crucial role in plant growth and development.

To date, the primary biochemical pathway of Chl degradation has been apparent based on the functional analysis of Chl catabolic genes. Due to the importance of pheophorbide *a* oxygenase (PAO) in Chl degradation, this pathway is referred to as PAO pathway (Christ et al. 2014; Kuai et al. 2018). Before Chl degradation, Chl *b* is converted to Chl *a* via two continuous enzymatic reactions calling chlorophyll cycling (Kusaba et al. 2007; Meguro et al. 2011). First, Chl *b* is reduced to 7-hydroxymethyl Chl *a* by Chl *b* reductase encoded by *NON*-*YELLOWCOLORING 1* (*NYC1*) and *NYC1*-*LIKE* (*NOL*) in *Arabidopsis*. Then 7-hydroxymethyl Chl *a* is converted to Chl *a* by 7- hydroxymethyl Chl *a* reductase (HCAR). Chl *a* is turned into a primary fluorescent Chl catabolite (*p*FCC) by four continuous steps. First, magnesium (Mg) in Chl *a* is removed by a Mg-dechelatase named STAY-GREENs (SGRs) in *Arabidopsis* and Chl *a* is converted into pheophytin *a* (Phein *a*) (Shimoda et al. 2016). Then Phein *a* is hydrolyzed to become pheophorbide *a* (Pheide *a*) and phytol by pheophytinase (PPH) (Schelbert et al. 2009). Along with the porphyrin ring of Pheide *a* being cleaved by PAO, the green color completely fades in Chl catabolite and Pheide *a* is converted to red Chl catabolite (RCC) (Pruzinska et al. 2003). Subsequently, RCC is catalyzed by red Chl actabolite reductase (RCCR) (Pruzinska et al. 2007) to become primary fluorescent Chl catabolite (*p*FCC) which is transferred out from chloroplasts and isomerized to non-fluorescent products by acidic pH in the vacuole (Christ et al. 2012; Hauenstein et al. 2016).

SGR is one of the most important enzymes in Chl degradation. The stay-green phenotype of *sgr* mutation was originally described by Mendel (1866). However, until 2007, the SGR homologs were initially identified in *Pisum sativum* (pea), *Arabidopsis*, and *Festuca pratensis* (Armstead et al. 2007). The mutation of SGR interferes with the normal senescence of leaves through affecting the Chl degradation; the senescing leaves in *sgr* mutants remain green for a long time. In the following decade, through mutants and transgenic technologies, SGR homologs were identified and analyzed in Chl degradation in a large number of species, for example, rice (Sato et al. 2007; Park et al. 2007; Jiang et al. 2007, 2011; Rong et al. 2013), *Arabidopsis* (Ren et al.^2007^; Aubry et al. 2008; Mecey et al. 2011; Sakuraba et al. 2012, 2014a, b), tomato (Barry et al. 2008; Hu et al. 2011; Luo et al. 2013), pepper (Barry et al. 2008; Borovsky and Paran 2008) and alfalfa (Zhou et al. 2011). However, in above studies, the SGR homologs in senescing chloroplasts only acted as recruiters for accelerating Chl degradation by interacting with five Chl catabolic enzymes (CCEs), NYC, HCAR, PPH, PAO, and RCCR in light-harvesting complex II (LHCII). Until 2016, it was found that SGR homologs played roles in Chl degradation as Mg-dechelatase in *Arabidopsis* and *Chlamydomonas reinhardtii*, respectively (Shimoda et al. 2016; Matsuda et al. 2016). Since then, all CCEs in the Chl degradation pathway of green plants have been identified, and the study of SGR homologs has entered a new field.

### Classification and sequence characteristic of SGR homologs

**Fig. 1:**
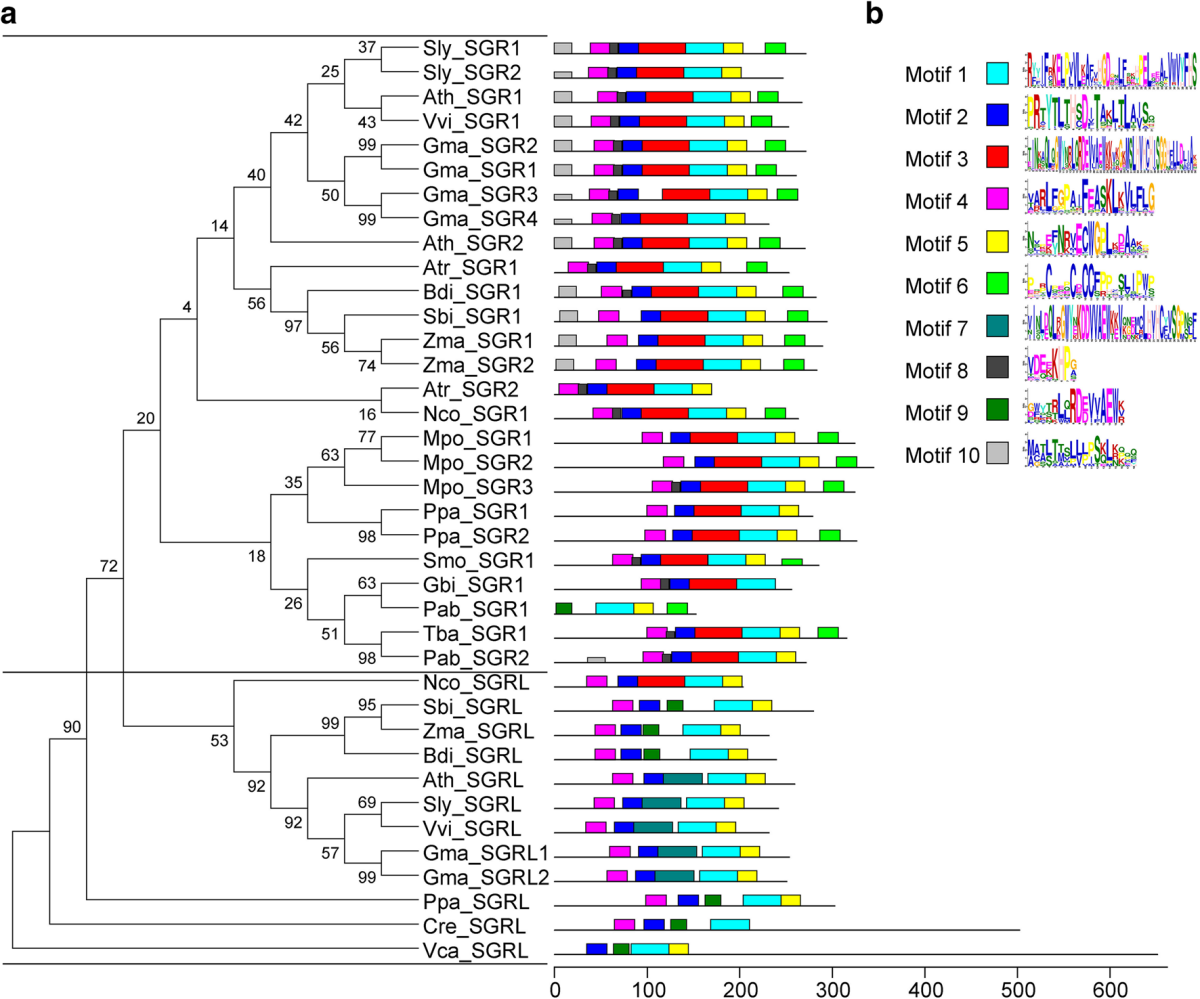
Phylogenetic tree and protein structure of SGR proteins. **a** Phylogenetic analysis of SGR proteins from various species. The phylogenetic tree of all sequences was constructed using Phylip 3.69 (http://evolution.gs.washington.edu/phylip.html) by the Neighbor-Joining (NJ) method, and with a bootstrap test with 1000 replications (Ming et al. 2020). The information of the SGR proteins is in Additional file 1: Table S1. **b** Conserved motifs of the SGR proteins were obtained using the MEME software (Zhang et al. 2018)

**Fig. 2:**
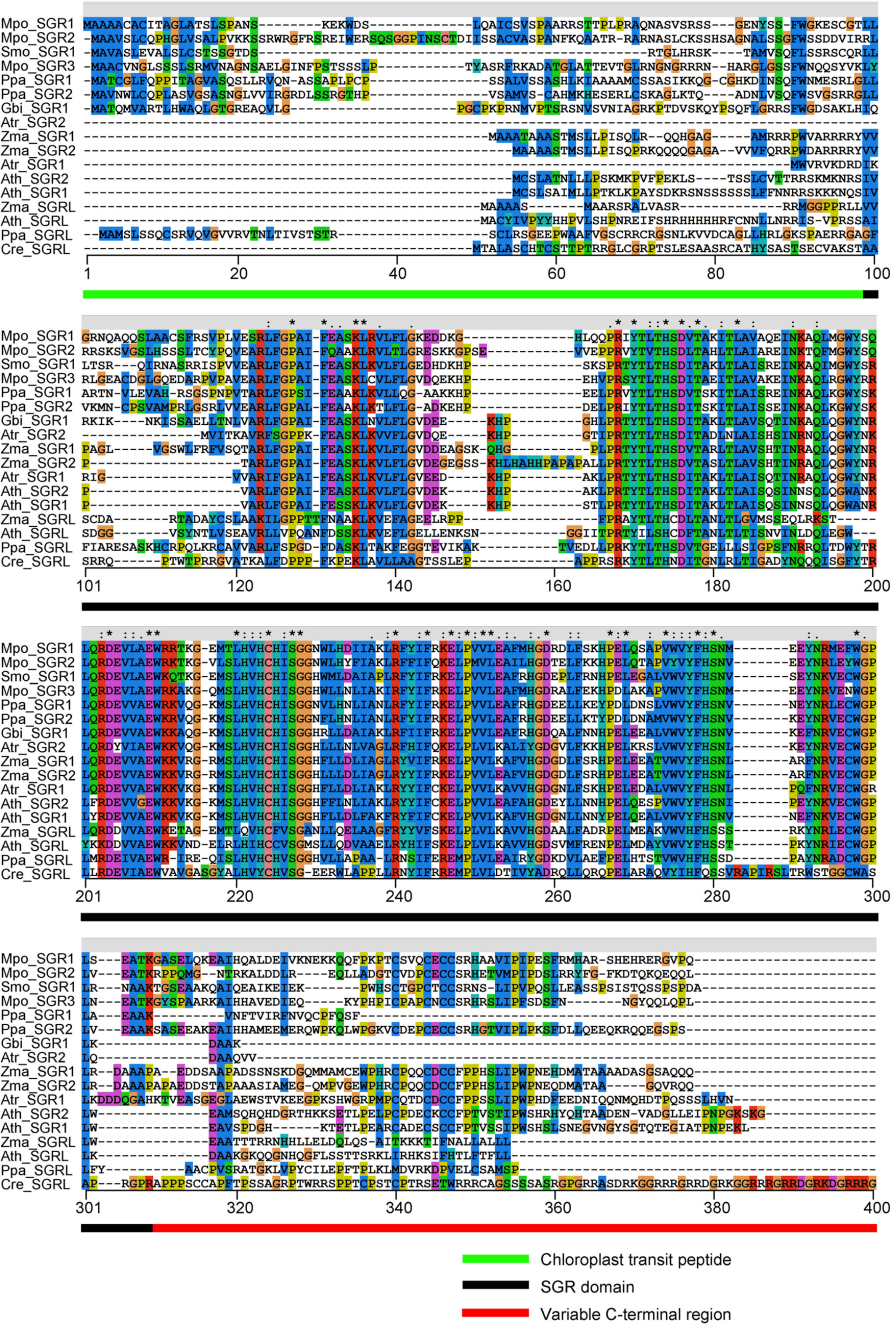
The multiple sequence alignment of SGR proteins. The multiple sequence alignment of SGR proteins was performed utilizing Clustal W 2.0.3 (http://www.ebi.ac.uk/clustalw/). The green, black, and red lines denote the chloroplast transit peptide, SGR domain, and variable C-terminal region. The information of the SGR proteins is in Additional file 1: Table S1

From *Chlamydomonas reinhardtii* (chlorophyta) to *Arabidopsis* (angiosperm), SGR homologs are widespread in the plant kingdom. Phylogenetic analysis reveals that there are two families of SGR homologs in plants, family I (SGR) and family II (SGRL) (Fig. 1a). The family I includes bryophytes, gymnosperms, and angiosperms. Multiple sequence alignment of the SGR homologs shows that they display a high degree of similarity (Fig. 2). All members are consisted of a highly conserved SGR domain, chloroplast transit peptide, and variable C-terminal region. The difference between SGR and SGRL lies in the C-terminal. SGR possesses a cysteine rich motif (CRM, P-X3-C-X3C-X-C2-F-P-X5-P), which is indispensable for Mg-dechelatase (Fig. 1b, motif6) (Xie et al. 2019), but there is not such motif in SGRL. Within the CRM motif, four cysteine residues are reported to participate in inter- or intramolecular crosslinking or in redox regulation. During natural senescence, a large amount of reactive oxygen species are produced, they lead to changes in redox potential in plants. These changes are conducive to the formation of dimer or polymer of SGR, and would therefore promote appropriately to the degradation of chlorophyll and detoxification during senescence. However, the CRM domain in SGRLs of land plants as well as in SGR of *Chlamydomonas reinhardtii* is absence, indicating that the CRM domain is obtained during the evolution of land plants. According to the evolutionary analyses, it can be seen that SGR homologs be originated from the green algae as early as possible in the course of evolution, which is consistent with its function in chlorophyll degradation. The differences in evolution result in SGR and SGRL two families, which may be the main reasons for the regulation of chlorophyll degradation in many aspects.

### Spatiotemporal expression of *SGR* homologs in *Arabidopsis*

**Fig. 3:**
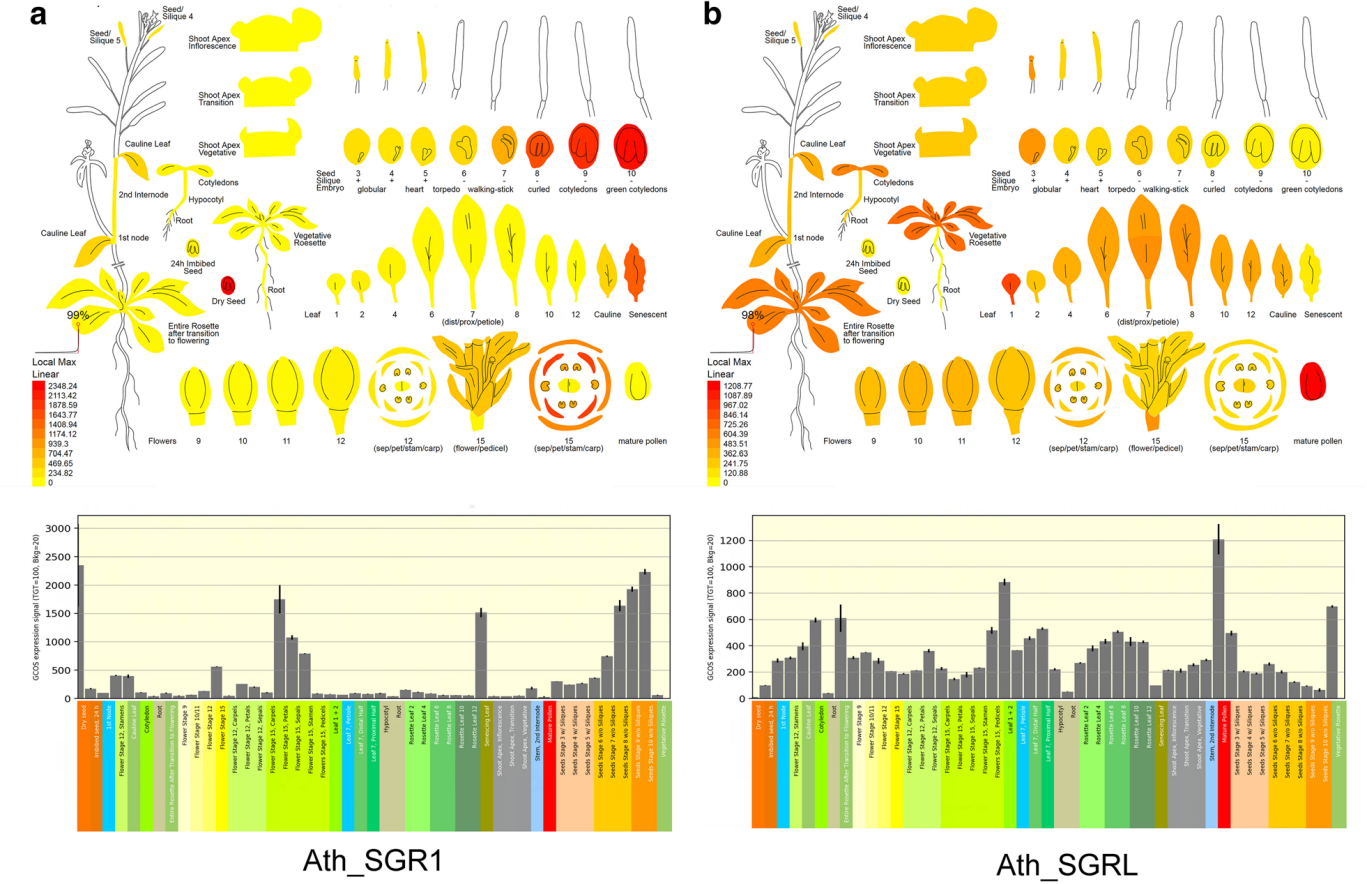
The spatiotemporal expression patterns of *SGR1* and *SGRL* in *Arabidopsis*. **a** The expression levels of *SGR1* in *Arabidopsis* in various tissues and at various development stages. **b** The expression levels of *SGRL* in *Arabidopsis* in various tissues and at various development stages

The function of genes is closely related to their spatiotemporal expression patterns. We analyzed the expression levels of *Arabidopsis SGR1* and *SGRL* in various tissues and at various development stages using the Arabidopsis eFP Browser (http://bar.utoronto.ca/) (Winter et al. 2007). As shown in Fig. 3, *SGR1* is mainly expressed in aging tissues, such as senescent leaves, seeds, and floral organs in the later stage of growth, and dry seeds, and the expression level of *SGR1* increases gradually with senescence of these tissues. This expression pattern indicated that SGR1 plays a vital role in chl degradation caused by natural senescence (Delmas et al. 2013; Li et al. 2017). However, the expression pattern of *SGRL* is opposite to that of *SGR1*. *SGRL* expresses in pre-senescing leaves, seeds, and floral organs, but its expression level decreases gradually with tissue aging. This implies that SGR1 and SGRL may function at different stages of plant growth and development. A previous study found that SGRL can perform similar functions to SGR1 in pre-senescing leaves under abiotic stress conditions, which possibly accelerates metabolic channeling of Chl breakdown intermediates to avoid accidental release of phototoxic Chl and Chl catabolites (Sakuraba et al. 2014b).

### The function of SGR homologs in Chl degradation

SGRs are chloroplast-localized proteins involving Chl degradation during leaf senescence (Hortensteiner 2009). The genetic screen of Chl degradation-disturbed mutants together with the subsequent isolation of responsible genes would greatly accelerate the elucidation of Chl degradation. In rice, the mutation of *SGR* results in the comparable expression of *NYC1* that encodes the first enzyme of Chl degradation as it in the wild-type plants (Sato et al. 2007). This finding suggests that SGR functions after the step of NYC1 in the Chl degradation pathway, which may be in the processes of translation or post-translation. In *Arabidopsis*, the research of a non-yellowing mutant *nye1*-*1* (*sgr1*) showed that there was a remarkable reduction of PAO activity, but no significant accumulation of either chlorophyllide *a* or Pheide *a* (Ren et al. 2007). Likewise, similar results were also found by Jiang et al. (2007) in rice. These results above collectively indicated that SGR1 played a crucial regulatory role in Chl degradation during senescence by modulating PAO activity. Chl-protein complexes in rice were more stable in *sgr* mutant than wide-type lines (Sato et al. 2007), suggesting that SGR not only promoted Chl degradation but also directly interacted with LHCII to accelerate the breakdown of Chl-protein complexes. In addition, it was found that SGR also promoted Chl degradation during fruit ripening in tomato and pepper (Borovsky and Paran 2008; Barry et al. 2008).

During leaf senescence of *Arabidopsis*, SGR1 could bind LHCII, and SGR and CCEs could also interact with each other at LHCII as demonstrated by yeast two-hybrid (Y2H) and bimolecular fluorescent complementary (BiFC) analyses (Sakuraba et al. 2012). These results indicated that SGR was essential for recruiting CCEs in senescing chloroplasts, and the SGR-CCEs-LHCII protein interaction would play an essential role in Chl degradation as well as Chl-protein complexes breakdown (Sakuraba et al. 2012). There were three homologs of SGR protein family in *Arabidopsis,* including SGR1, SGR2, and SGR-like (SGRL). Similarly to SGR1 during leaf senescence, expression of *Arabidopsis* SGR2 was highly up-regulated, but the function of SGR2 counteracted SGR1 activity during leaf senescence (Sakuraba et al. 2014a). In contrast to SGR1, during senescence, *SGR2*-overexpressing lines stayed green and the *sgr2*-*1* mutant exhibited early leaf yellowing. Through Y2H, Co-immunoprecipitation (Co-IP), and pull-down assays, it was revealed that SGR2 interacted with LHCII and SGR1 but with other CCEs hardly (Sakuraba et al. 2014a). So it strongly suggested that SGR2 maybe played a negative regulatory role in Chl degradation by possibly interfering with the CCEs-recruiting function of SGR1 (Sakuraba et al. 2014a). Interestingly, the data of Wu et al. (2016) demonstrated that SGR2 played a positive regulatory role in Chl degradation, which was contrary to the previous result (Sakuraba et al. 2014a). For example, the authors did not observe the early leaf yellowing phenotype in *nye2*-*1* (Aubry et al. 2008) and the stay-green phenotype in *SGR2* overexpression lines (Sakuraba et al. 2014a). Therefore, it was suggested that the phenotypic discrepancies should be identified furtherly and SGR2 may function as an assistant paralog of SGR1, which was upregulated in the absence of SGR1 (Wu et al. 2016). These discrepancies might be caused by genetic effects or distinct growth conditions. In addition to SGR1 and SGR2, SGRL was one of the important SGR homologs that functioned to accelerate Chl degradation under abiotic stress. For example, in *Arabidopsis*, overexpressing *SGRL* showed early leaf yellowing, while *sgrl*-*1* mutant exhibited stay-green phenotype in leaves. Under salt stress, SGRL could rapidly degrade Chl and Chl-protein complexes synergistically with SGR1 through forming homo- or heterodimers and interacting with LHCII and CCEs (Sakuraba et al. 2014b). In rice, SGRL could promote Chl degradation in dark-induced senescence (Rong et al. 2013). In sum, SGR homologs involved in Chl degradation, but the relationship between them and the role of SGRL under abiotic stress should be discussed in development.

In the last decade, the function of SGR homologs during Chl degradation and the interaction between SGR homologs and other CCEs had been demonstrated. However, the molecular mechanism of SGRs in Chl breakdown has not yet been illuminated. In 2016, the Mg-dechelatase activity of SGR homologs was convincingly found through the wheat germ protein expression system in *Arabidopsis* (Shimoda et al. 2016) and *Chlamydomonas reinhardtii* (Matsuda et al. 2016), respectively. Researches on *Arabidopsis* showed that SGR1/2 could extract Mg from Chl *a* but had very low or no activity against chlorophyllide *a*. On the contrary, SGRL could have higher activity against chlorophyllide *a* than Chl *a*, and all SGRs could not extract Mg from Chl *b*. Moreover, enzymatic experiments using the light-harvesting complexes revealed that SGRs could extract Mg also from the Chl-protein complexes (Shimoda et al. 2016). In addition to *Arabidopsis*, it was found that Phein *a* increased after the incubation of Chl *a* with CrSGR of *Chlamydomonas reinhardtii* in *E. coli*, but Chl *b* and 7-hydroxymethyl Chl *a* did change little or no when incubating with CrSGR in *E. coli*. Similar to *Arabidopsis*, these observations also showed that Chl *a* was the most suitable substrate for CrSGR (Matsuda et al. 2016). The finding that SGRs serve as Mg-dechelatase in green plants supplemented the pathway of Chl degradation. At the same time, the results that SGRs acted after the reduction reaction of Chl *b* and before the formation of Phein *a* (Aubry et al. 2008). Chen and his colleagues gave these studies that SGRs acted as Mg-dechelatase a good evaluation in the review (Chen et al. 2016). They proposed that clarifying the exact roles of SGR homologs as Mg-dechelatase resolved a long-lasting mystery and especially filled the significant gap in the Chl breakdown pathway, which would greatly promote the future researches of the elaborate regulation of Chl catabolism. The conversion of Chl *a* to Phein *a* by SGRs was the first and crucial regulatory step during Chl degradation. Remarkably, Phein *a* was an essential molecule in photosystem (PS) II, so SGRs might affect the formation of PSII. Despite the SGRs in *Arabidopsis* and *Chlamydomonas reinhardtii* having the same catalytic property, the physiological functions of SGRs were diverse (Chen et al. 2018). For example, SGRs in *Arabidopsis* took part in Chl degradation while SGRs in *Chlamydomonas reinhardtii* participated in PSII formation (Chen et al. 2018). Although the recent studies have demonstrated that SGRs are able to remove Mg from Chl *a* to initiate its breakdown, little is known about the domain basis of its functionality. Xie et al. showed that in *Arabidopsis* there were conserved cysteine-rich motifs (CRM: P-X3-C-X3C-X-C2-F-P-X5-P) at C terminus of SGR1 and SGR2, but not in SGRL. Genetic analysis and enzymatic assays demonstrated that all four cysteines in the CRM played irreplaceable roles in the conformational change and Mg-dechelating activity (Xie et al. 2019).

### Other function of SGR homologs

SGRs not only play crucial roles during Chl degradation in leaf senescence but also function in other organs during plant growth and development. Loss of both SGR1 and SGR2 in *Arabidopsis* results in nearly complete retention of Chl during leaf senescence and green seeds (Delmas et al. 2013). Further studies of Li et al. (2017) revealed that an over-accumulation of free Chl caused serious photo-damage during seed maturation, because of a burst of reactive oxygen species. Taken together, these data above proposed that efficient SGRs-mediated Chl degradation was significant for detoxification during seed maturation.

All SGR homologs from algae to higher plants were predicted to be localized in chloroplasts (Xie et al. 2019), revealing that they likely acted in plastids, most likely during Chl degradation. So as one of the CCEs, it was easy to understand that seeds of *sgr* mutants exhibited stay-green phenotype. And, interestingly, the SGR1 in tomato also plays a crucial role during fruit maturation. It was found that SGR1 could change the accumulation pattern of lycopene through directly interacting with PSY1 that was a key enzyme in the carotenoid metabolism pathway by inhibiting its activity. Moreover, SGR1 alters the expression of ethylene-induced genes and ethylene receptor genes, thus may involve in ethylene signaling. Therefore, in SGR1-repressed tomato lines, the fruit shelf-life was prolonged obviously (Luo et al. 2013). Based on these results, one intriguing question was prompted about whether SGR1 regulated carotenoid accumulation in other tissues, such as senescent leaves, or other plant species. Because of declining in the level of carotenoids following Chl degradation during leaf senescence (Biswal 1995), it was proposed that carotenoid degradation pathways were activated and carotenoid biosynthesis pathways were repressed during leaf senescence. Thus, SGR1 could play a pivotal role in the regulation of carotenoid metabolism.

Except for the role in carotenoid biosynthesis, SGR in *Medicago truncatula* (MtSGR) is also involved in nodule development and senescence (Zhou et al. 2011). Expression of *MtSGR* was detected in all nodules zones and higher than any other organs, including senescent leaves. This study indicated that several nodule senescence associated genes were significantly down-regulated in the nodules of *Medicago truncatula sgr* mutants (termed NF2089), and indicated that MtSGR affects nodule senescence in legumes. Functional analyses of SGR1 in fruit ripening of tomato and MtSGR in nodule senescence of *Medicago truncatula* were the intriguing evidence that SGRs not only functioned in chloroplasts but also generally acted in other plastids and executed their multiple functions. Based on the above results, our understanding of the underlying roles of SGRs will be expanded in plant growth and development processes beyond Chl degradation.

### Transcriptional regulatory of SGR homologs

Chl breakdown is one of the most significant characteristics in the course of leaf senescence and fruit ripening, which is regulated by multiple internal and external signals, such as phytohormones and environmental cues. SGR homologs, as the first enzymes for initiating Chl *a* degradation, are also regulated by various factors, especially abscisic acid (ABA).

ABA, as a plant hormone, plays a positive role in leaf senescence and Chl degradation. Three ABA-responsive element (ABRE) binding transcription factors, ABF2 (AREB1), ABF3, and ABF4 (AREB2), were identified as the putative transcription factors binding SGR1 promoter in *Arabidopsis* by yeast one-hybrid (Y1H) screening. Furthermore, they were demonstrated to directly bind the *SGR1* promoter and consequently activate the expression of *SGR1* gene. The triple mutant of the *ABFs*, *abf2abf3abf4*, as well as two SGR mutants, *sgr1* and *sgr2* exhibited stay-green phenotypes during leaf senescence upon ABA treatment, along with reduced the expression of *SGR1* and *SGR2*. In contrast, overexpression of *ABF4* could induce the expression of *SGR1* and *SGR2* to accelerate Chl breakdown after ABA treatment. This suggested that ABF2, ABF3, and ABF4 likely functioned as key positive regulators in mediating ABA-triggered Chl degradation and leaf senescence through activating the expression of *SGRs* in *Arabidopsis* (Gao et al. 2016). Apart from ABFs, ABSCISIC ACID INSENSITIVE 5 (ABI5), a key transcription factor in the ABA signaling pathway, was also demonstrated to function as a positive regulator of *SGR1* via binding the ABRE motif in the promoter of *SGR1*. Consistent with the above results, in *abi5* mutants, the expression level of *SGR1* was significantly downregulated (Sakuraba et al. 2014c). In mature seeds, Chl non-degradation was an undesirable trait influencing seed maturation, seed oil quality, and meal quality. The researchers found that ABA-dependent transcription factor ABSCISIC ACID INSENSITIVE 3 (ABI3) could directly bind to the *SGR1* promoter and activate its expression. Electrophoretic mobility shift assay (EMSA) showed that the B3 domain of ABI3 could bind to the RY motif (CATGCA) in the promotors of *SGR1* and *SGR2* (Delmas et al. 2013).

## Conclusion

After years of effort, the study on plant SGR homologs has made significant progress, but there is still a lot of ambiguity in the regulative mechanism of SGRs. The related researches focus on *Arabidopsis* and other model plants, and the multiple functions of SGRs in other plants require to be illuminated further. Since the identification of the first SGR protein in pea, the functions are discovered extensively from the recruiters interacting directly or indirectly with CCEs to the Mg-dechelatase, via multiple biological techniques such as genetic engineering and bioinformatics. However, the understanding of the regulative mechanism of SGRs involved in Chl breakdown is still unclear. The perspective above of SGR homologs might provide clues for further research. In sum, in-depth research of the regulative mechanism of SGR homologs in Chl degradation and plant senescence will be a hot research area in the future. At the same time, the “stay-green” trait may be a focal point of future breeding projects because its potential to improve crop quality and yield.

## Supplementary information

**Additional file 1: Table S1.** The information of all SGR family sequences.

## Data Availability

Not applicable.

## Abbreviations

ABA: Abscisic acid
ABI3: ABSCISIC ACID INSENSITIVE 3
ABRE: ABA-responsive element
Chl: Chlorophyll
CCEs: Chl catabolic enzymes
CRM: Cysteine-rich motifs
HCAR: 7-hydroxymethyl chlorophyll a reductase
LHCII: Light-harvesting complex II
NOL: NYC1-LIKE
NYC1: NON-YELLOW COLORING 1
PAO: Pheophorbide *a* oxygenase
*p*FCC: Primary fluorescent Chl catabolite
Phein *a*: Pheophytin *a*
Pheide *a*: Pheophorbide *a*
PPH: Pheophytinase
RCC: Red Chl catabolite
RCCR: Red Chl actabolite reductase
SGR: STAY-GREEN
SGRL: SGR-LIKE
