# Supplementary material for: Roles of stay-green (SGR) homologs during chlorophyll degradation in green plants
Source: Bot Stud. 2020 Sep 23;61:25. doi: 10.1186/s40529-020-00302-5 (PMC7511501; doi:10.1186/s40529-020-00302-5)
Supplement: Supplementary file 1 — Additional file 1: Table S1. The information of all SGR family sequences. [file 40529_2020_302_MOESM1_ESM.docx]

Table S1. The information of all SGR family sequences

| Name | NO. | Source | Specie name |
| --- | --- | --- | --- |
| Cre_SGRL | XP_001690907.1 | https://www.ncbi.nlm.nih.gov/ | *Chlamydomonas reinhardtii* |
| Vca_SGRL | XP_002948199.1 | https://www.ncbi.nlm.nih.gov/ | *Volvox carteri* |
| Ppa_SGR1 | PNR58080.1 | https://www.ncbi.nlm.nih.gov/ | *Physcomitrella patens* |
| Ppa_SGR2 | XP_024399918.1 | https://www.ncbi.nlm.nih.gov/ | *Physcomitrella patens* |
| Ppa_SGRL | XP_024383632.1 | https://www.ncbi.nlm.nih.gov/ | *Physcomitrella patens* |
| Mpo_SGR1 | OAE35361.1 | https://www.ncbi.nlm.nih.gov/ | *Marchantia polymorpha* |
| Mpo_SGR2 | OAE25837.1 | https://www.ncbi.nlm.nih.gov/ | *Marchantia polymorpha* |
| Mpo_SGR3 | PTQ31267.1 | https://www.ncbi.nlm.nih.gov/ | *Marchantia polymorpha* |
| Smo_SGR1 | XP_002968798.2 | https://www.ncbi.nlm.nih.gov/ | *Selaginella moellendorffii* |
| Gbi_SGR1 | GBI00007833 | https://bioinformatics.psb.ugent.be/plaza/ | *Ginkgo biloba* |
| Tba_SGR1 | TBA00006505 | https://bioinformatics.psb.ugent.be/plaza/ | *Taxus baccata* |
| Pab_SGR1 | PAB00011941 | https://bioinformatics.psb.ugent.be/plaza/ | *Picea abies* |
| Pab_SGR2 | PAB00052561 | https://bioinformatics.psb.ugent.be/plaza/ | *Picea abies* |
| Nco_SGR1 | XP_031493988.1 | https://www.ncbi.nlm.nih.gov/ | *Nymphaea colorata* |
| Nco_SGRL | XP_031477920.1 | https://www.ncbi.nlm.nih.gov/ | *Nymphaea colorata* |
| Atr_SGR1 | ERM96319.1 | https://www.ncbi.nlm.nih.gov/ | *Amborella trichopoda* |
| Atr_SGR2 | ERN08737.1 | https://www.ncbi.nlm.nih.gov/ | *Amborella trichopoda* |
| Bdi_SGR1 | XP_003576766.1 | https://www.ncbi.nlm.nih.gov/ | *Brachypodium distachyon* |
| Bdi_SGRL | XP_003580904.1 | https://www.ncbi.nlm.nih.gov/ | *Brachypodium distachyon* |
| Sbi_SGR1 | XP_002462718.1 | https://www.ncbi.nlm.nih.gov/ | *Sorghum bicolor* |
| Sbi_SGRL | XP_002448084.2 | https://www.ncbi.nlm.nih.gov/ | *Sorghum bicolor* |
| Zma_SGR1 | ONM56739.1 | https://www.ncbi.nlm.nih.gov/ | *Zea mays* |
| Zma_SGR2 | NP_001105770.2 | https://www.ncbi.nlm.nih.gov/ | *Zea mays* |
| Zma_SGRL | NP_001130909.1 | https://www.ncbi.nlm.nih.gov/ | *Zea mays* |
| Sly_SGR1 | NP_001234723.1 | https://www.ncbi.nlm.nih.gov/ | *Solanum lycopersicum* |
| Sly_SGR2 | XP_004252642.1 | https://www.ncbi.nlm.nih.gov/ | *Solanum lycopersicum* |
| Sly_SGRL | XP_004237702.1 | https://www.ncbi.nlm.nih.gov/ | *Solanum lycopersicum* |
| Gma_SGR1 | NP_001238357.1 | https://www.ncbi.nlm.nih.gov/ | *Glycine max* |
| Gma_SGR2 | NP_001236690.1 | https://www.ncbi.nlm.nih.gov/ | *Glycine max* |
| Gma_SGR3 | XP_003550881.2 | https://www.ncbi.nlm.nih.gov/ | *Glycine max* |
| Gma_SGR4 | XP_003549871.1 | https://www.ncbi.nlm.nih.gov/ | *Glycine max* |
| Gma_SGRL1 | XP_003523416.1 | https://www.ncbi.nlm.nih.gov/ | *Glycine max* |
| Gma_SGRL2 | NP_001351655.1 | https://www.ncbi.nlm.nih.gov/ | *Glycine max* |
| Ath_SGR1 | NP_001328989.1 | https://www.ncbi.nlm.nih.gov/ | *Arabidopsis thaliana* |
| Ath_SGR2 | NP_192928.2 | https://www.ncbi.nlm.nih.gov/ | *Arabidopsis thaliana* |
| Ath_SGRL | NP_564489.1 | https://www.ncbi.nlm.nih.gov/ | *Arabidopsis thaliana* |
| Vvi_SGR1 | CBI34771.3 | https://www.ncbi.nlm.nih.gov/ | *Vitis vinifera* |
| Vvi_SGRL | CBI18940.3 | https://www.ncbi.nlm.nih.gov/ | *Vitis vinifera* |
